# Effect of High-Quality Whole-Course Care on Psychological Status and Postoperative Pharyngeal Complications in Patients Undergoing Surgery for Hyperparathyroidism Secondary to Chronic Rrenal Failure

**DOI:** 10.3389/fsurg.2022.905413

**Published:** 2022-05-19

**Authors:** Qinghong Luo, Shuquan Zheng

**Affiliations:** ^1^ The Nanhua Affiliated Hospital, Department of Thyroid and Breast Surgery, Hengyang Medical School, University of South China, Hengyang, China; ^2^ The Nanhua Affiliated Hospital, Department of Hemodialysis Room, Hengyang Medical School, University of South China, Hengyang, China

**Keywords:** chronic renal failure, secondary hyperparathyroidism, high-quality whole-course care, psychological status, surgery, pharyngeal complications

## Abstract

**Objective:**

To observe the effects of high-quality whole-course care on the psychological status and postoperative pharyngeal complications in patients undergoing surgery for secondary hyperparathyroidism (SHPT) to chronic rrenal failure (CRF).

**Methods:**

The clinical data of 62 patients who underwent surgical treatment for CRF-SHPT from April 2018 to October 2021 in our department were retrospectively analyzed. According to the different nursing methods after admission, they were divided into two groups, of which 33 patients who received high-quality whole-course care were the high-quality group, and 29 patients who received routine nursing were the regular group. Compliance, occurrence of pharyngeal complications, improvement of preoperative and postoperative psychological status [Assessed by self-rating anxiety scale (SAS) and self-rating depression scale (SDS)], nursing satisfaction scores, and serum hormone levels [intact parathyroid hormone (iPTH), calcium (Ca), Phosphorus (P)] were compared between the two groups.

**Results:**

The differences between the general conditions and clinical characteristics of the two groups were not significant (*p* > 0.05). After care, the number of cases with good compliance in the high-quality group was higher than that in the regular group, and the number of cases with non-compliance was lower than that in the regular group (*p* < 0.05); the difference in the number of cases with partial compliance after care between the two groups was not significant (*p* > 0.05). There was no significant difference in the incidence of pharyngeal complications such as sore throat, nausea and vomiting, dry throat and hoarseness between the two groups (*p* > 0.05); however, the 24-h postoperative sore throat and dry throat scores in the high-quality group were significantly lower than those in the regular group (*p* < 0.05). Patients in the high-quality group had higher nursing attitude, nursing skills, nursing safety, nursing quality, and overall nursing satisfaction scores than the regular group (*p* < 0.05). Compared with the pre-care period, SAS and SDS scores decreased in both groups after care, and SAS and SDS scores decreased more in the high-quality group than in the regular group (*p* < 0.05). Serum iPTH, Ca, and P levels decreased in both groups at 1 week after surgery, and iPTH, Ca, and P levels decreased more in the high-quality group than in the regular group (*p* < 0.05).

**Conclusion:**

Through the high-quality whole-course care, full informed participation and active cooperation of CRF-SHPT patients, close medical and nursing collaboration, attention to detail and overall level of treatment can effectively improve patient compliance, psychological status and postoperative serum indicators, promote patient recovery and improve nursing satisfaction.

## Preface

Secondary hyperparathyroidism (SHPT) is a series of symptoms caused by excessive production and secretion of parathyroid hormone by the parathyroid glands due to chronic renal insufficiency, renal tubular acidosis, vitamin D deficiency, Fanconi syndrome, digestive system reactions, pregnancy and lactation (1, 2). Among the various factors that trigger this disease, SHPT due to chronic renal failure (CRF) is the most common and is an important factor affecting the quality of survival of patients with CRF (3). Medical drug therapy and hemodialysis are currently the main treatment methods for CRF-SHPT patients, but about 50% of them still cannot relieve the clinical symptoms of SHPT after medical drugs or hemodialysis, and can gradually progress to refractory or progressive disease, at which point the patient will require surgical treatment (4, 5). However, these patients undoubtedly increase the risk of surgery due to long-term hemodialysis and combined multisystemic pathologies, such as impaired coagulation mechanisms, hypoproteinemia, and renal anemia (6). In addition, the operation itself is a stressful event for the patient, which often brings greater psychological pressure to the patient. and the patient is prone to negative emotions such as anxiety and depression, which in turn have a negative impact on the patient’s postoperative regression (7), so it is necessary to strengthen all aspects of patient care after admission. High-quality whole-course care (8, 9) is a nursing service for the entire surgical treatment process, implemented according to the characteristics of preoperative, intraoperative and postoperative work, so that the patient is in an optimal state at the physical, psychological and spiritual levels.

Due to the complexity of CRF-SHPT patients, numerous perioperative complications and difficult management, the authors have conducted a study on the effect of High-quality care of CRF-SHPT patients throughout the whole process since 2018, and achieved better results in the perioperative management of CRF-SHPT patients.

### Information and Methods

#### Case Collection

The clinical data of 62 patients who underwent surgical treatment for CRF-SHPT in our department from April 2018 to October 2021 were retrospectively analyzed. Patients were admitted to the hospital and were treated surgically, according to the different nursing methods after admission, they were divided into two groups, of which 33 patients who received high-quality whole-course care were the high-quality group, and 29 patients who received routine nursing were the regular group.

#### Data Review

General clinical data including gender, age, weight, age on dialysis, and other general demographic information were collected from the subjects. All subjects were tested for serum calcium, phosphorus, and whole segment parathyroid hormone (iPTH) before surgery and 1 week after surgery.

#### Inclusion Criteria

① Patients with indications for surgery (10), such as (i) joint and bone pain and/or skeletal deformities, significant symptoms of muscle weakness, myalgia and skin pruritus; (ii) persistent blood iPTH > 800 ng/L with hypercalcemia or hyperphosphatemia; (iii) Color Doppler ultrasound showed at least 1 or more enlarged parathyroid glands >1 cm in diameter with abundant blood flow; (iv) Failure of internal medicine treatment.
② No severe skeletal deformity and osteoporosis, severe coagulation dysfunction, or combined with serious systemic diseases such as heart, lung, and brain dysfunction.
③ ASA classification of grade I to III.
④ Aged 20–65 years.

#### Exclusion Criteria

① Patients with recent history of upper respiratory tract infection, cough, or throat discomfort.
② Previous history of pharyngeal surgery.
③ Primary parathyroid disease.
④ Psychiatric disorders, etc., and the presence of physical disabilities.
⑤ The patient or the patient’s family gave informed consent to this treatment plan.

### Study Methods

#### Regular Group

##### Post-admission to Preoperative

Preoperatively, the nursing staff closely observed the patient’s blood pressure, heart rate, pulse and other vital signs.

##### Intraoperatively

Nursing staff should actively cooperate with the physician, closely observe the patient’s temperature, pulse and other relevant changes, and promptly notify the physician for treatment if any situation arises. In terms of diet, the patient should be advised to pay attention to diet and daily living habits, and take medication regularly.

##### Postoperative to Hospital Discharge

The patient was placed in a flat position and nursing staff closely monitored the patient’s skin and pulse; if abnormalities occurred, they were recorded and the physician was notified in a timely manner; when anti-infective drugs were administered, the patient’s condition was closely monitored and the physician was notified in a timely manner if abnormalities occurred.

#### High-Quality Group

##### The High-Quality Whole-Course Care Responsibility Group was Established

The members of the quality treatment responsibility group all received detailed training on quality treatment and knowledge related to CRF-SHPT surgery, and developed scientific and reasonable quality treatment measures.

##### Quality Management Measures

The treatment problems were determined with regard to the etiology and surgical characteristics of CRF-SHPT, the causes of major perioperative complications, prevention and treatment points, and the specific conditions of patients.

##### Post-Admission to Preoperative

① Psychological counseling was carried out: As patients suffered from long-term disease as well as lifelong dialysis and expensive medical costs, they were generally in a state of tension and depression before surgery; coupled with a lack of confidence in the upcoming surgery and fear of surgical risks and prognosis, they were more prone to varying degrees of fear and anxiety, so psychological counselling should be carried out. ② Hypercalcemia management: Monitored the patient’s serum Ca, P level, when the patient has nausea and vomiting symptoms, should be alert to the occurrence of critical signs. When the patient’s blood Ca was greater than 3.2 mmol/L, he (she) should be promptly given a low-calcium diet and drink more water, drinking >1,500 mL/d. Daily intake and output were recorded to maintain the balance of output, and blood potassium was monitored to prevent hypokalemia due to massive urination; when the patient’s blood calcium was >3.75 mmol/L, the patient should be resuscitated as hypercalcemic, regardless of the manifestation of hypercalcemic crisis. ③ Preoperative preparation: the last preoperative dialysis was performed with heparin-free or low-molecular heparin dialysis to reduce intraoperative and postoperative bleeding. Trained patients to adapt to shoulder elevation and neck hyperextension position to ensure smooth operation, and patients were instructed to pay attention to safety when moving around, not to fall, prevent falling from bed and avoid fracture.

##### Intra-Operative

Helped the anesthesiologist to do a good job of anesthesia, and when the anesthesia was successful, we had to position the patient to a safe and comfortable angle, and take 2 mL venous blood specimens for the patient before, 5 minutes and 10 minutes after surgery, and send them to the laboratory for PTH testing. The changes in the patient’s blood oxygen saturation, pulse, blood pressure, heart rate and other indicators were closely observed during the operation. The infusion rate should be strictly controlled, and once the patient is found to have hand and foot convulsion symptoms, 10–20 mL of calcium gluconate (concentration of 10%) should be slowly pushed intravenously in a timely manner.

##### Postoperative to Hospital Discharge

① Strict observation of vital signs: For postoperative patients, vital signs should be strictly observed, especially if there was a tendency of bleeding during dialysis or hypertension with heart failure, notify the doctor immediately and gave symptomatic treatment. Kept a tracheotomy kit at the patient’s bedside for 24 h after surgery, and resuscitated the patient in time if he/she was found to have respiratory distress or tendency to suffocate. ② Control of infection: Due to the large amount of toxins that accumulate in the body of CRF-SHPT patients and cannot be eliminated on their own, the water-electrolyte disorders in the body were highly susceptible to infectious diseases such as skin mucosal infections and stomatitis. Some studies concluded that active oral care and skin care were the keys to preventing pressure sores and infections, while ventilating, disinfecting and reducing visits to the ward. Monitor blood count, blood sugar and kidney function at all times so that problems could be notified to the doctor in time for appropriate treatment. ③ Health education was provided to patients and their families to guide them to choose a nutritious diet and to enhance the intake of iron-containing foods. It was important to avoid consuming iron-containing foods while quoted tea and coffee to avoid affecting the absorption of iron in the body, but you could increase vitamin C-rich vegetables and fruits at the same time with iron-containing foods to promote their absorption.

### Observed Indicators

#### Compliance

The classification of compliance of surgical patients was divided into 3 levels: ① Good compliance: Perioperative patients fully accepted the treatment plan formulated by the physician, complete the complete treatment process, and fully cooperate with all care measures. ② Partial compliance: Perioperative patients accepted the treatment plan developed by their physicians and partially cooperated with the nursing measures. ③ Non-compliance: Perioperative patients did not understand the reactions and side effects that occur and do not cooperate with medical care..

#### Post-Operative Complications

The occurrence, severity and incidence of sore throat, dry throat, nausea and vomiting, and hoarseness at 24 h after surgery were recorded in the two groups. ① Postoperative sore throat: the degree was evaluated by numerical rating system (NRS) (11): 0 being no pain, and 10 being the most painful. ② Dry throat score: A score of 0 indicated that the patient felt no difference from before surgery and had no symptoms of dry throat. 1 indicated mild discomfort, the patient felt different from before surgery and had symptoms of dry throat, but it was not obvious that there was no feeling of stuffiness and/or constriction and no foreign body sensation in the pharynx. A score of 2 indicated moderate discomfort, mainly dryness of the throat with a feeling of stuffiness and/or constriction, with minimal self-consciousness, no or no foreign body sensation in the throat, occasional throat clearing, no habitual throat clearing, and a score of 3 indicated severe discomfort, with a constant habitual throat clearing to ensure the comfort of the throat, a feeling of stuffiness and/or constriction, and a feeling of reduced mucus secretion in the throat and an obvious foreign body sensation in the throat.

#### Nursing Satisfaction

A questionnaire designed in-house was used to determine the satisfaction level of nursing care for both groups. The questionnaire was scored on a percentage scale, and the nursing satisfaction score consisted of 20 items in four dimensions: nursing attitude, nursing skills, nursing safety and nursing quality. Assessments were conducted separately before and after care and were rated by the same assessor.

#### Mental Status

Self-rating anxiety scale (SAS) and self-rating depression scale (SDS) were used to determine the severity of patients’ anxiety and depression negative emotions were positively correlated with the scores (12). A final SAS score of <50 indicates no anxiety, with higher scores indicating more severe anxiety. A final SDS score of <53 indicates no depression, with higher scores indicating more severe depression. Assessments were conducted separately before and after care and were rated by the same assessor.

#### Serological Indicators

The serum iPTH, Ca and P levels of the patients were compared preoperatively and at the 1st postoperative week. Serum iPTH was measured by enzyme-linked immunosorbent assay (The relevant kits were purchased from Shanghai mlbio Co.), and serum Ca and P were measured by fully automated biochemical analyzer(Shandong Boke Biological Industry Co.).

### Statistical Methods

SPSS 20.0 was used for data analysis, and the independent sample t-test or Kruskal-Wallis and Mann-Whitney method tests were used for measurement data, and Fisher’S exact probability method was used for count data. *P* < 0.05 was considered a statistically significant difference.

## Results

### General and Clinical Characteristics

General clinical data of the subjects were collected, including general demographic data and clinical characteristics such as gender, age, height, years on dialysis, and ASA classification, and iPTH, Ca, and P were tested before care. The results of the analysis showed that there was no significant difference between patients in the regular and high-quality group s in terms of gender, age, years on dialysis, weight, preoperative iPTH, Ca, and P levels, and the ratio of ASA classification, intractable pruritus, bone pain, insomnia, muscle weakness, and restless leg syndrome (*p* > 0.05) (Table 1).

**Table 1:** General condition level clinical characteristics of patients before care.

Information		Regular group (*n* = 29)	High-quality group (*n* = 33)	t or χ^2^ value	*p* value
Sex (Male, %)		16 (55.17)	20 (60.61)	0.187	0.665
Age (years; Mean, SD)		46.56 ± 8.45	47.02 ± 8.73	0.210	0.834
Years of dialysis (years; Mean, SD)		7.16 ± 3.46	7.01 ± 3.22	0.177	0.860
Body weight (kg; Mean, SD)		61.80 ± 9.42	62.17 ± 9.93	0.150	0.881
ASA classification (*n*, %)	Grade II	11 (37.93)	14 (42.42)	0.130	0.719
	Grade III	18 (62.07)	19 (57.58)		
	Intractable pruritus	9 (31.03)	11 (33.33)	0.037	0.847
	Bone pain	20 (68.97)	18 (54.55)	1.353	0.245
Clinical features (*n*, %)	Insomnia	7 (24.14)	9(24.24)	0.079	0.778
	Myasthenia	16 (55.17)	19 (57.58)	0.036	0.849
	Restless legs syndrome	10 (34.48)	11 (33.33)	0.009	0.924
iPTH (ng/L)		1,738.50 ± 726.20	1,746.36 ± 733.53	0.042	0.966
Ca (mmol/L)		2.76 ± 0.25	2.70 ± 0.27	0.753	0.454
P (mmol/L)		2.30 ± 0.40	2.28 ± 0.31	0.221	0.826

### Adherence

The results of the pre-care measurement showed that there was no significant difference in the distribution of the three levels of good, partial and non-adherence between the regular group and the high-quality group (*p* > 0.05). After care, the number of cases of good compliance was higher, and the number of cases of non-compliance was lower in the high-quality group when compared to the regular group (*p* < 0.05); the difference in the number of cases of partial compliance between the two groups after care was not significant (*p* > 0.05) (Table 2).

**Table 2:** Comparison of compliance before and after care between the two groups (*n*, %).

Group	Good compliance		Partially compliant		Non-compliant	
	Before care	After care	Before care	After care	Before care	After care
Regular group (*n* = 29)	8 (27.59)	10 (34.48)	13 (44.83)	12 (41.38)	8 (27.59)	7 (24.13)
High-quality group (*n* = 33)	10 (30.30)	21 (63.64)*	16 (48.48)	11 (33.33)	7 (21.21)	1 (3.03)*
χ^2^ value	0.098	5.248	0.083	0.428	0.342	6.119
*p-*value	0.754	0.022	0.773	0.513	0.559	0.013

*Note:* *Shows the difference with the same group before care, p < 0.05.

### Throat Complications

**Figure 1:**
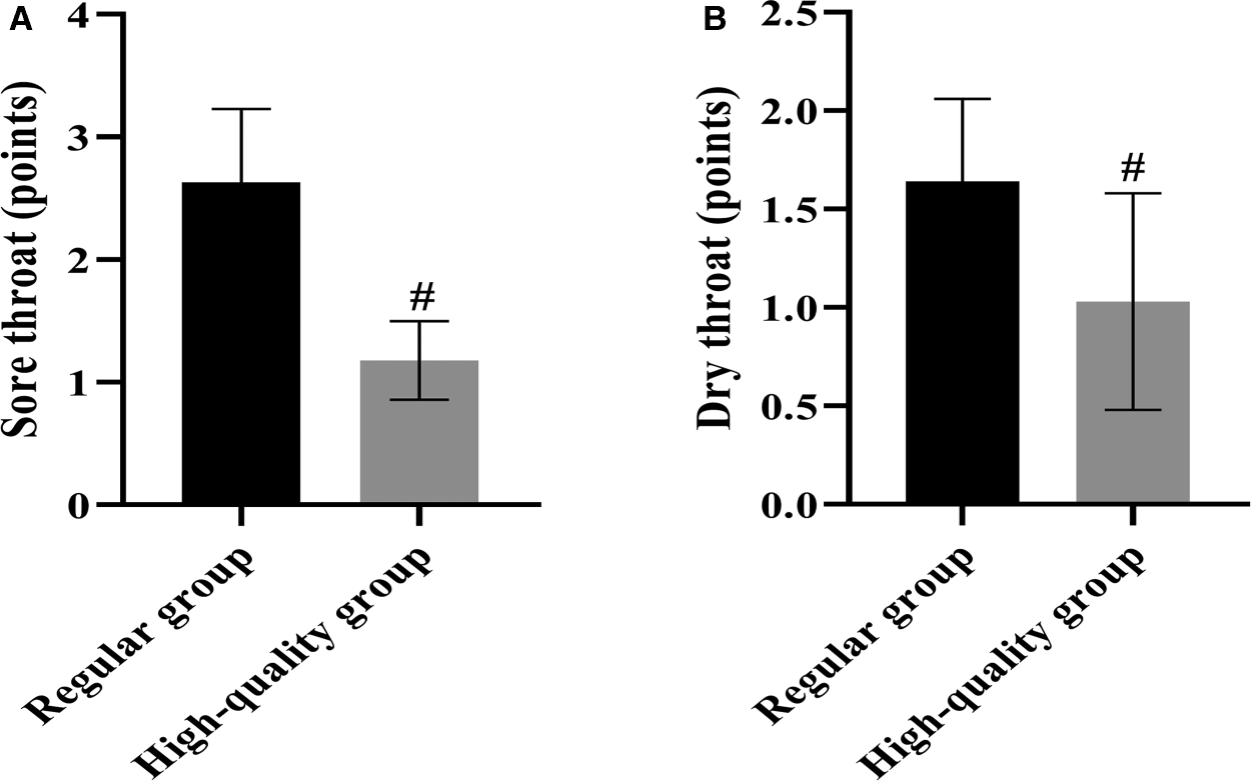
Comparison of the severity of postoperative pharyngeal complications between the two groups (Mean, SD). Note: (**A**) shows the sore throat score and (**B**) shows the dry throat score. # shows the difference with the Regular group, *p* < 0.05.

We observed and counted the occurrence of pharyngeal complications such as sore throat, nausea and vomiting, dry throat, and hoarseness in the regular group and the high-quality group at 24 h postoperatively, and scored the degree of sore throat and dry throat. The results of the analysis showed that there was no significant difference in the incidence of pharyngeal complications such as sore throat, nausea and vomiting, dry throat and hoarseness between the two groups (*p* > 0.05); however, the scores of sore throat and dry throat in the high-quality group were significantly lower than the regular group 24 h after surgery (*p* < 0.05) (Table 3 and Figure 1).

**Table 3:** Comparison of the occurrence of postoperative pharyngeal complications between the two groups (*n*,%).

Group	Sore throat	Nausea and vomiting	Dry throat	Hoarseness
Regular group (*n* = 29)	4 (13.79)	4 (13.79)	3 (10.34)	2 (6.90)
High-quality group (*n* = 33)	3 (9.09)	2 (6.06)	1 (3.03)	0 (0.00)
χ^2^ value	0.341	1.056	1.368	2.272
*p-*value	0.559	0.304	0.242	0.132

### Nursing Satisfaction

**Figure 2:**
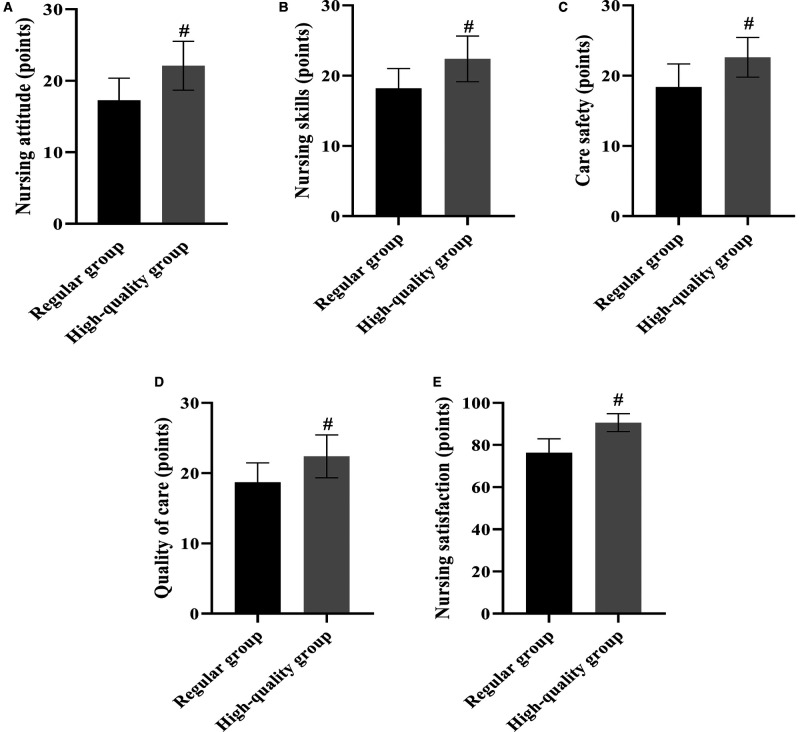
Comparison of various scores of nursing satisfaction after care in the two groups (Mean, SD). Note: (**A**) shows nursing attitude scores, (**B**) shows nursing skill scores, (**C**) shows nursing safety scores, (**D**) shows quality of care scores, and E shows total nursing satisfaction scores. # shows the difference with the Regular group, *p* < 0.05.

The degree of patient satisfaction with nursing care in both groups was determined after nursing care using our in-house designed questionnaire. The results of the analysis showed that the patients in the high-quality group had higher ratings of nursing attitude, nursing skills, nursing safety, nursing quality, and overall nursing satisfaction than the regular group (*p* < 0.05) (Figure 2).

### Pre- and Post-Care Psychological Status Scores

**Figure 3:**
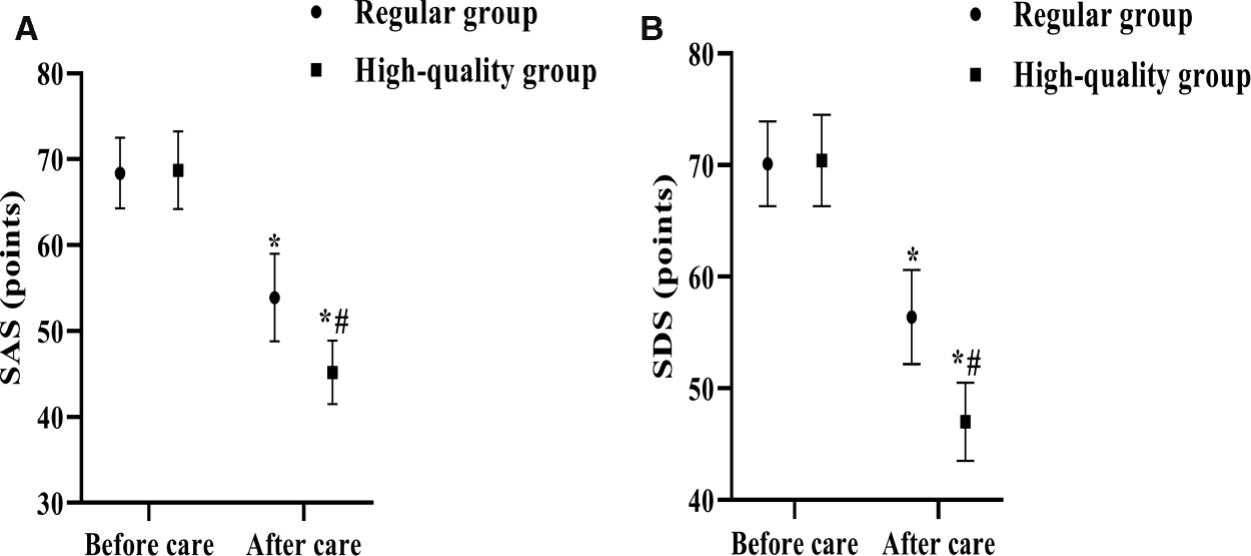
Comparison of psychological status scores between the two groups before and after care. (Mean, SD), Note: (**A**) shows SAS scores, (**B**) shows SDS scores. * shows the difference with the same group before care, *p* < 0.05; # shows the difference with the Regular group, *p* < 0.05.

The SAS and SDS scales were used to assess the patients’ anxiety and depression before and after care. The results of the analysis showed that the difference between the SAS and SDS scores of the regular group and the high-quality group before care was not significant (*p* > 0.05). After care, the SAS and SDS scores of both groups decreased, and the SAS and SDS scores of the high-quality group decreased more than the regular group (*p* < 0.05) (Figure 3).

### Serological Indicators

**Figure 4:**
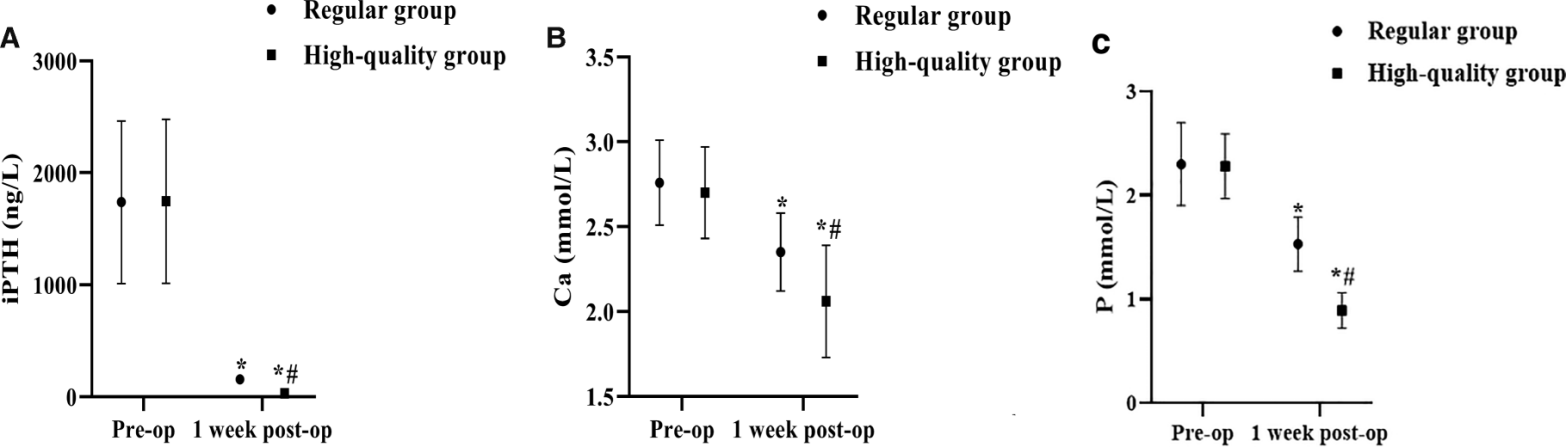
Comparison of iPTH, Ca, and P levels between the two groups before and 1 week after surgery. (Mean, SD). Note: (**A**) shows iPTH levels, (**B**) shows Ca levels, and (**C**) shows P levels. * shows the difference with the same group before care, *p* < 0.05; # shows the difference with the Regular group, *p* < 0.05.

Serum iPTH, Ca, and P levels were measured pre-op and 1 week post-op, respectively. The results of the analysis showed that the differences between the pre-op serum iPTH, Ca and P levels of the regular group and the high-quality group were not significant (*P* > 0.05). Serum iPTH, Ca, and P levels in both groups decreased at 1 week post-op, and the iPTH, Ca, and P scores in the high-quality group decreased more than those in the regular group (*p* < 0.05) (Figure 4).

## Discussion

SHPT is one of the common serious complications in CRF patients, especially in patients on long-term maintenance dialysis therapy. In CRF, the stimulation of the parathyroid glands by the imbalance of acid-base balance and various electrolyte metabolism disorders, as well as the presence of calcium and phosphorus metabolism disorders and active vitamin D deficiency in the middle and late stages of renal failure promote the secretion of PTH and induce the development of SHPT (13, 14). Studies have shown (15) that hypersecretion of PTH and hyperplasia and hypertrophy of parathyroid glands are characteristic of hyperparathyroidism. Patients with CRF-SHPT tend to present with hypercalcemia, osteoporosis, bone pain, skeletal deformities and pathological fractures, and most patients come to the clinic when they develop persistent bone pain (16). In China, because there are fewer kidney sources available for kidney transplantation and more patients with chronic renal impairment to chronic renal failure due to various reasons, patients with chronic renal failure are treated with hemodialysis or peritoneal dialysis with drug treatment for complications in order to maintain the quality of life of these patients and prolong their life span (17, 18). Over the past 20 years, SHPT has been well controlled due to the development of dialysis technology and early intervention with drugs, but some patients with refractory SHPT still require surgical treatment. However, the condition of these patients is complex, with many complications, and the surgical treatment of general anesthesia with tracheal intubation is an invasive operation, which can easily cause local nerve compression, airway mucosal injury, and vocal cord injury; the damage to the pharyngeal mucosa caused by tracheal intubation can lead to an imbalance in the ratio of the attached local flora, and some bacteria can move down with the catheter, which can increase the risk of infection, and then form harmful irritation and increase the risk of pharyngeal complications such as sore throat and dry throat (19, 20). Therefore, more effort needs to be invested before and after the procedure in CRF-SHPT patients to enable them to safely survive the perioperative period.

In this study, by observing the effect of implementing full quality care for patients undergoing CRF-SHPT surgery, we found that patient compliance, satisfaction with care, and improvement in SAS and SDS scores after care were better in the high-quality group than in the regular group. Although there was no significant difference in the incidence of pharyngeal complications between the two groups at 24 h postoperatively, the sore throat and dry throat degree scores were lower in the high-quality group than in the regular group. This suggests that the whole process of quality care can effectively improve the compliance of patients undergoing CRF-SHPT surgery, reduce patients’ bad mood, make patients accept the surgery in a good state of mind, and better cooperate with the treatment and nursing work; it can also reduce the degree of sore throat and dry throat of patients 24 h after surgery to accelerate their postoperative recovery, and improve the recognition and satisfaction of nursing work. In addition, the serum iPTH, Ca, and P levels of patients in the high-quality group were lower than those in the regular group at 1 week postoperatively, suggesting that conventional care enables faster correction of calcium and phosphorus disorders in patients undergoing CRF-SHPT. High-quality whole-course care takes quality care as the main nursing concept and enhances the efficiency of nursing work with a patient-centered service concept in the nursing process. By establishing a professional full quality nursing team, the nursing staff’s level of care has been enhanced to provide a comfortable treatment environment for patients undergoing CRF-SHPT, which is conducive to enhancing the comfort level of the patient’s organism, thereby increasing patient compliance with treatment (21, 22). Preoperative quality treatment was carried out mainly for patients’ psychological stress, hypercalcemia and surgical adaptation exercises, and medical and nursing education measures were further strengthened to give patients a more comprehensive understanding of the purpose, steps and procedures, and effects of the upcoming surgery, to fully prepare them psychologically, to eliminate their nervousness and concerns, and to reasonably avoid surgical risks (23, 24). Care through intraoperative sign monitoring, position selection, encouragement support and temperature maintenance can ensure a smooth operation. By planning a proper diet and actively taking good oral care and skin care after surgery, patients can reduce the discomfort level of sore throat, dry throat and other throat complications.

## Conclusion

In conclusion, this study confirms that through the whole process of quality case, fully informed participation and active cooperation of CRF-SHPT patients, close medical and nursing collaboration, attention to detail and overall level of treatment can effectively improve patient compliance, psychological status and postoperative serum indicators, promote patient recovery and increase nursing satisfaction.

## Data Availability

The original contributions presented in the study are included in the article/Supplementary Material, further inquiries can be directed to the corresponding author/s.
